# Efficacy of paraspinal muscle-sparing approach in the treatment of thoracolumbar burst fractures with neurological deficits

**DOI:** 10.12669/pjms.42.5.12988

**Published:** 2026-05

**Authors:** Binsong Tang, Zheng Lei, Fan Li, Taiheng Liu, Quanxiang Liu

**Affiliations:** 1 Binsong Tang Department of Orthopaedics, The Affiliated Hospital of Bei-hua University, Jilin 132011, Jilin, China; 2 Zheng Lei Department of Orthopaedics, The Third Bethune Hospital of Jilin University, Changchun 130021, Jilin, China; 3 Fan Li Department of Orthopaedics, The Affiliated Hospital of Bei-hua University, Jilin 132011, Jilin, China; 4 Taiheng Liu Department of Orthopaedics, Jilin people’s Hospital, Jilin 132011, Jilin, China; 5 Quanxiang Liu Department of Orthopaedics, The Affiliated Hospital of Bei-hua University, Jilin 132011, Jilin, China

**Keywords:** Paraspinal muscle-sparing approach, Thoracolumbar, Burst fracture, Neurological deficit

## Abstract

**Objective:**

To evaluate the clinical efficacy of pedicle screw fixation via a paraspinal muscle-sparing approach in the surgical treatment of thoracolumbar burst fractures(TCBFs) complicated by neurological deficits.

**Methodology:**

A total of 90 patients with TCBFs and associated neurological deficits, treated at The Affiliated Hospital of Bei-hua University between January 2024 to January 2025 were retrospectively analyzed. These patients were divided into a posterior midline approach(PMA) group, paraspinal muscle-sparing approach(PMSA) group and minimally invasive percutaneous approach(MIPA) group by the choice of surgical approach. Compared perioperative parameters, postoperative recovery, pre- and post-operative Cobb angles and anterior vertebral body height of the fractured segments among the three groups.

**Results:**

All surgical incisions healed primarily without infection and there was no cases of implant failure during the one-year follow-up. Significant differences were found among the three groups in duration of DoO, IBL, PDV and LoHS (all *P<* 0.05). Postoperative improvements in the Cobb angle and relative anterior height of the fractured vertebra were significant within each group, but no statistically significant differences were observed among the three groups(all *P>* 0.05). VAS scores significantly decreased over time in all groups, while ADL and JOA scores progressively increased, with within-group differences at different time points reaching statistical significance(all *P<* 0.05), no statistically significant intergroup differences were observed in VAS, ADL, or JOA scores at corresponding time points(all *P>* 0.05).

**Conclusion:**

Pedicle screw fixation via a paraspinal muscle-sparing approach is a safe and effective treatment for TCBFs with neurological deficits, which can facilitate radiographic correction and promote postoperative neurological recovery.

## INTRODUCTION

The thoracolumbar vertebral region constitutes the most heavily loaded segment of the spine and exhibits substantial mobility. Thoracolumbar fractures are among the most common types of spinal injuries, typically characterized by marked instability and frequently accompanied by neurological deficits, which can severely compromise patients’ physical health and quality of life.^1^ At present, surgical intervention remains the mainstay of treatment for thoracolumbar fractures. Considering its favorable clinical outcomes, the conventional open posterior midline approach is preferred. However, this technique necessitates extensive dissection and prolonged retraction of the paraspinal musculature, resulting in substantial surgical trauma and partial disruption of the spine’s normal anatomical structures. Consequently, patients may experience persistent postoperative back pain, adversely affecting long-term prognosis.^2^

In recent years, minimally invasive percutaneous and paraspinal muscle-sparing approaches have emerged as alternative surgical strategies. The percutaneous approach involves the placement of working cannulas through soft tissue corridors, thereby minimizing dissection and disruption of the paraspinal muscles. The paraspinal muscle-sparing approach involves inserting pedicle screws through the natural intermuscular plane between the multifidus and longissimus muscles, an anatomical space with relatively sparse neurovascular structures. This approach requires only blunt separation of the paraspinal musculature to adequately expose the surgical field, thereby facilitating operative manipulation.^3^ Both minimally invasive techniques significantly reduce paraspinal muscle dissection and prolonged traction, preserve physiological spinal structures, lower the risk of postoperative complications and promote functional recovery.

Despite the growing adoption of these techniques, few studies have systematically compared the outcomes of the posterior midline approach, the minimally invasive percutaneous approach and the paraspinal muscle-sparing approach in the management of thoracolumbar burst fractures (TCBFs), particularly in cases complicated by neurological deficits. Against this backdrop, a retrospective observational study was conducted on these techniques in the clinical treatment of TCBFs with associated neurological deficits, aiming to evaluate and compare the clinical efficacy and practical value of the paraspinal muscle-sparing approach in this context.

## METHODOLOGY

A total of 90 patients with TCBFs complicated by neurological deficits who were admitted to The Affiliated Hospital of Bei-hua University between January 2024 to January 2025 were retrospectively enrolled in this study. Based on the surgical approach, patients were divided into three groups, namely the paraspinal muscle-sparing approach (PMSA) group, the posterior midline approach (PMA) group and the minimally invasive percutaneous approach (MIPA) group.

### Ethical Approval:

The study was approved by the Institutional Ethics Committee of The Affiliated Hospital of Bei-Hua University (No.2022-09-18; Dated: December 01, 2024) and written informed consent was obtained from all participants.

### Inclusion criteria:

- Single-level TCBFs.
- Fresh fractures.
- Presence of neurological impairment.
- Written consent to surgical treatment.

### Exclusion criteria:

- Contraindications to surgery.
- Refusal of surgical treatment.
- Old or chronic thoracolumbar fractures.
- Patients with underlying diseases such as diabetes and coronary heart disease.
- Pathological fractures.

### Surgical Procedure:

Patients in the PMSA group underwent surgery via the paraspinal muscle-sparing approach. Under general anesthesia, the patient was placed in the prone position. After standard sterilization and draping, the fractured vertebral segment was localized and marked using C-arm fluoroscopy. A midline posterior incision was made centering on the fractured vertebra and the soft tissues were dissected layer by layer to expose the lumbodorsal fascia. The fascia was incised longitudinally 1.5-2.5 cm lateral to the spinous process. Blunt dissection was then performed through the natural intermuscular plane between the longissimus and multifidus muscles to expose the facet joints and transverse processes. Pedicle screws were inserted into the injured vertebra and the adjacent vertebrae.

Following fluoroscopic confirmation of proper screw placement, pre-contoured rods were inserted and distraction was applied to reduce the fractured vertebra. If necessary, particularly in cases with neurological compromise or canal intrusion, a fenestration was made on the lamina at the side of neurological deficit or bony intrusion. The affected nerve root was decompressed and displaced bony fragments within the spinal canal were repositioned using an “L”-shaped retractor. The bone graft bed was enlarged and autologous or allogeneic bone grafts were implanted. Final fluoroscopy was performed to confirm adequate fracture reduction and absence of compressive material within the spinal canal. The screws were tightened, the wound was irrigated and a drainage tube was placed. The incision was closed in layers and covered with sterile dressings to complete the procedure. Patients in the PMA group underwent internal fixation via the conventional open posterior midline approach.

Under general anesthesia, patients were placed in the prone position. Following fluoroscopic localization of the fracture segment, standard disinfection and sterile draping were performed. A posterior midline incision approximately 15-20 cm in length was made. The skin, subcutaneous tissue and thoracolumbar fascia were sequentially incised. The bilateral paraspinal muscles were detached along both sides of the spinous processes to fully expose the posterior spinal elements. The remaining procedures were performed in accordance with those in the PMSA group. Patients in the MIPA group underwent surgery via the minimally invasive percutaneous approach. Under general anesthesia, the patient was positioned prone and standard sterile preparation was conducted.

The fractured vertebral segment was identified using the same fluoroscopic technique as applied in the PMSA group. The percutaneous puncture sites were then determined and guidewires were inserted. Centered on each guidewire, a 1.5-2.5 cm skin incision was made. The skin and subcutaneous tissues were incised and working sleeves were inserted over the guidewires with sequential dilation to establish the working channels. With the assistance of the working cannulas, appropriately sized cannulated pedicle screws were inserted step-by-step. Fluoroscopy was used to confirm optimal screw placement. Pre-bent connecting rods were then inserted and fracture reduction was achieved using a distractor. In cases with neurological involvement or spinal canal compromise, the incision was appropriately extended on the side of neurological deficit or bony intrusion. A laminotomy was performed, followed by nerve root decompression and bone grafting. Final fluoroscopy was conducted to verify satisfactory fracture reduction and ensure that no residual compressive elements remained within the spinal canal. The screws were tightened, the wound was irrigated and a drainage tube was placed. The incision was closed in layers and covered with sterile dressings to complete the procedure. The follow-up work of all patients was completed by the same group of surgeons, and patients in both groups received the same standard of nursing intervention.

### Outcome Measures:

- *Perioperative Parameters:* The following perioperative parameters were recorded and compared among the three groups: duration of operation (DoO), intraoperative blood loss (IBL), postoperative drainage volume (PDV) and length of hospital stay (LoHS).
- *Imaging Parameters:* The Cobb angle and relative anterior vertebral body height of the fractured vertebra were measured preoperatively and at one year postoperatively in all three groups. The Cobb angle was defined as the angle between the superior endplate of the vertebra above the fracture and the inferior endplate of the vertebra below the fracture. Relative anterior height (RAH) of the fractured vertebra was calculated as: RAH = Actual anterior height / Reference anterior height) × 100%.
- *Pain Intensity and Neurological Function:* Visual Analog Scale (VAS), Activities of Daily Living (ADL) and Japanese Orthopaedic Association (JOA) scores were documented and compared among the three groups at three time points: preoperatively, six months postoperatively and one year postoperatively, to assess pain intensity and neurological recovery.
- *Clinical Outcomes and Complications:* Clinical efficacy at one year postoperatively was evaluated using the modified MacNab criteria. Complications occurring during the follow-up period, including surgical site infection, internal fixation failure (*e.g*., implant breakage, loosening, or displacement) and cerebrospinal fluid (CSF) leakage, were recorded and compared among the three groups.

### Statistical Analysis:

All statistical analyses were performed using SPSS 22.0. Normality of distribution for continuous variables was assessed prior to further analysis. Data conforming to a normal distribution were expressed as mean ± standard deviation (). One-way analysis of variance was used for intergroup comparisons of continuous variables. Categorical variables were presented as frequencies and percentages (*n*[%]) and compared using the chi-square (χ²) test. A *p*-value of < 0.05 was considered statistically significant.

## RESULTS

Based on the inclusion and exclusion criteria, the PMSA group included 30 patients (19 males and 11 females) aged between 34 and 74 years, with a mean age of 57.47 ± 9.42 years. Injury causes were traffic accidents in 11 cases, falls in 15 cases and others in four cases. Fracture levels involved the thoracic spine in 14 cases and the lumbar spine in 16 cases. The PMA group consisted of 30 patients (22 males and eight females) aged between 37 and 73 years, with a mean age of 57.50 ± 8.94 years. Causes of injury were traffic accidents in 12 cases, falls in 13 cases and others in five cases. Fracture levels included 18 thoracic and 12 lumbar vertebrae. The MIPA group comprised 30 patients (17 males and 13 females) aged between 37 and 78 years, with a mean age of 58.40 ± 8.58 years. Injuries were caused by traffic accidents in 12 cases, falls in 14 cases and other accidents in four cases. Fractures involved the thoracic spine in 16 cases and the lumbar spine in 14 cases. There were no statistically significant differences in baseline characteristics among the three groups (all *P >* 0.05), indicating good comparability Table-I.

**Table-I:** Comparison of baseline characteristics among the three groups (n = 30).

Group	Sex (M/F)	Age (years)	Cause of Injury (Traffic accident/Fall/Others)	Fracture Level (Thoracic/Lumbar)
PMSA	19/11	57.47±9.42	11/15/4	14/16
PMA	22/8	57.50±8.94	12/13/5	18/12
MIPA	17/13	58.40±8.58	12/14/4	16/14
*F/χ²-value*	1.843	0.104	0.354	1.071
*P-value*	0.398	0.901	0.986	0.585

Significant differences were observed among the three groups in DoO, IBL, PDV and POHS (all *P <* 0.05). The PMSA group had the shortest DoO, while the PMA group had the longest. The MIPA group exhibited the least IBL and PDV, whereas the PMA group had the most. Both the PMSA and MIPA groups had shorter LoHS compared with the PMA group Table-II.

**Table-II:** Comparison of perioperative outcomes between groups (*x̄*+*s*).

Group	DoO (min)	IBL (mL)	PDV (mL)	LoHS (d)
PMSA	92.17±7.39	216.17±8.68	92.33±17.36	6.83±1.02
PMA	133.67±7.65	289.33±8.28	147.33±23.33	8.17±1.82
MIPA	109.33±5.53	138.00±9.61	84.67±9.73	6.63±0.81
*F-value*	272.31	2182.048	111.835	12.481
*P-value*	0.000	0.000	0.000	0.000

Preoperatively, there were no statistically significant differences among the three groups in the Cobb angle or RAH (*P >* 0.05, respectively). Postoperatively, all three groups showed significant improvement in both parameters, indicating effective fracture reduction and spinal realignment; however, no statistically significant intergroup differences were observed in the degree of improvement (all *P >* 0.05) Table-III.

**Table-III:** Comparison of pre- and post-operative Cobb angle and RAH (*x̄*+*s*).

Group	Preoperative Cobb angle (°)	Postoperative Cobb angle (°)	Preoperative RAH (%)	Postoperative RAH (%)
PMSA	22.23±0.34	6.74±2.34	48.36±7.23	91.23±6.27
PMA	22.37±0.42	7.42±2.00	49.23±7.49	91.66±6.82
MIPA	22.25±0.45	7.47±2.33	47.93±6.96	88.74±6.70
*F-value*	1.113	1.005	0.253	1.704
*P-value*	0.333	0.370	0.777	0.188

VAS scores in all three groups decreased significantly over time, while ADL and JOA scores increased progressively. Within-group comparisons at different time points revealed statistically significant differences (*P <* 0.05, respectively). However, no statistically significant intergroup differences were observed in VAS, ADL, or JOA scores at any single time point (all *P >* 0.05) Table-IV.

**Table-IV:** Comparison of VAS, ADL and JOA scores at different time points (*x̄*+*s*).

Group	Time Point	VAS score	ADL score	JOA score
PMSA	Pre-op	7.53±0.51	45.40±2.61	10.27±3.44
	At 6 months post-op	3.57±0.50	71.53±3.19	19.63±2.97
	At one year post-op	1.33±0.66	84.40±3.59	24.10±2.17
PMA	Pre-op	7.30±0.47	44.57±3.34	10.93±2.94
	At 6 months post-op	3.70±0.60	72.47±4.07	19.97±2.70
	At one year post-op	1.53±0.63	85.87±5.24	23.43±2.01
MIPA	Pre-op	7.47±0.51	45.10±3.71	9.93±3.19
	At 6 months post-op	3.37±0.49	70.47±3.49	18.37±2.44
	At one year post-op	1.20±0.66	84.17±3.70	22.80±2.09

At one year follow-up, the rates of excellent and good outcomes according to the modified MacNab criteria were 90.00% in the PMSA group, 86.67% in the PMA group and 80.00% in the MIPA group. The differences among the three groups were not statistically significant (*P >* 0.05). Table-V. With all wounds achieving primary healing, no surgical site infection was observed in any group. During the one-year follow-up period, no cases of internal fixation failure (e.g., implant breakage, loosening, or displacement) were reported.

**Table-V:** Comparison of modified MacNab outcomes at one year follow-up (n[%]).

Group	n	Excellent	Good	Moderate	Poor	Excellent + Good (%)
PMSA	30	20(66.67)	7(23.33)	2(6.67)	1(3.33)	27(90.00)
PMA	30	18(60.00)	8(26.67)	3(10.00)	1(3.33)	26(86.67)
MIPA	30	15(50.00)	9(30.00)	5(16.67)	3(10.00)	24(80.00)
*χ² value*						1.259
*P-value*						0.533

## DISCUSSION

In this study, we compared perioperative parameters, imaging parameters and clinical outcomes among the three surgical groups. The results showed that, compared with the conventional posterior midline approach, both the paraspinal muscle-sparing approach and the minimally invasive percutaneous approach significantly reduced IBL, DoO, PDV and LoHS. These findings indicate that minimally invasive approaches can improve perioperative outcomes, enabling early mobilization and rehabilitation training. Early mobilization helps prevent muscle stiffness and atrophy, thereby promoting clinical recovery. In terms of imaging parameters, all three groups demonstrated significant improvements in thoracolumbar Cobb angles for kyphosis and the RAH of the fractured vertebra postoperatively. However, no significant differences were observed among the three groups, suggesting that the corrective effects of the minimally invasive approaches are comparable to those of the conventional posterior midline approach and all three techniques can effectively restore vertebral alignment and function. No serious complications occurred in any of the groups during treatment or follow-up and clinical outcomes did not differ significantly among the groups. Improvements in postoperative pain and neurological function were observed across all groups, with no statistically significant differences at each follow-up time point. These results suggest that both the paraspinal muscle-sparing and minimally invasive percutaneous approaches are as effective as the conventional posterior midline approach in alleviating pain and promoting neurological recovery. Same with previous reports,^4^,^5^ which have confirmed that, compared with the conventional posterior midline approach, both the paraspinal and percutaneous approaches are associated with reduced surgical trauma and improved postoperative outcomes.

In recent years, clinical data have indicated a marked increase in the incidence of thoracolumbar fractures, with TCBFs representing one of the most severe forms.^6^,^7^ These injuries typically involve fractures of the anterior and middle spinal columns,^8^ with retropulsed bony fragments protruding into the spinal canal. The displacement of these fragments frequently leads to neurological impairment, particularly involving the cauda equina and spinal cord, thereby increasing the complexity of clinical management. Previous studies have demonstrated a significant correlation between the extent of spinal neurological impairment and the sagittal diameter of the spinal canal.^9^ Chen HW*et al*.^10^ found that patients with TCBF-induced neural canal compromise are more prone to develop neurological deficits. Additionally, injuries involving the posterior spinal elements were associated with more pronounced spinal cord dysfunction. However, it is noteworthy that, in such cases, neurological function may recover rapidly following appropriate intervention. These findings underscore the importance of selecting timely, appropriate and effective treatment strategies for this patient population. To date, there remains some controversy regarding the optimal management of TCBFs. Several studies have reported that conservative treatment may also yield satisfactory outcomes in selected cases.^11^ Nonetheless, the prevailing view supports surgical intervention, particularly in patients with TCBFs or neurological deficits, in order to achieve spinal decompression and restore stability.^12^ Early surgical intervention not only facilitates neurological recovery but also significantly reduces the incidence of fracture- and surgery-related complications and mortality.^13^

The surgical treatment of TCBFs can be broadly classified into anterior and posterior approaches. Specifically, posterior fixation with pedicle screws combined with neural decompression is a standard procedure commonly used for fracture stabilization and spinal fusion in TCBFs. However, the conventional open posterior approach requires extensive dissection and prolonged retraction of the paraspinal muscles during surgery. This excessive manipulation can result in ischemic necrosis and denervation of the paraspinal musculature, leading to postoperative complications such as persistent low back pain and rigid deformity, which may significantly compromise surgical outcomes.^14^

Additionally, during pedicle screw insertion, paraspinal muscle bulk may obscure the surgical field and interfere with the optimal angle and accuracy of screw placement.^15^ To minimize iatrogenic injury to the paraspinal muscles and improve the precision of pedicle screw placement, various alternative surgical approaches have been explored. Among them, the paraspinal muscle-sparing approach and the minimally invasive percutaneous approach have gained the most success and are now widely adopted in clinical practice.^16^ The paraspinal muscle-sparing approach was first proposed by Watkins in 1959.^17^ Subsequent modifications were introduced by Wiltse, who refined the technique into the widely known Wiltse approach. This approach involves a posterior midline skin incision carried down to the lumbodorsal fascia, followed by bilateral dissection through the natural intermuscular plane between the longissimus and multifidus muscles to expose the facet joints. Under direct visualization, pedicle screws are then inserted into the vertebrae.^18^ The minimally invasive percutaneous approach involves making four paramedian skin incisions, each approximately 1.5 cm in length, in the lumbar region. Working cannulas are inserted through these incisions and surgical instruments are manipulated through the cannulas to complete the procedure.^19^ Studies have demonstrated that both the paraspinal muscle-sparing approach and the minimally invasive percutaneous approach significantly reduce muscle dissection and traction of the paraspinal musculature, offering the advantages of minimally invasive surgery and promoting better postoperative recovery.^20^

### Limitations:

It includes the retrospective analysis based on small sample size, no long-term follow-up observation, which was limited, so the conclusions drawn may not be very convincing. Further analysis with expanded samples and variables is necessitated to further verify potential efficacy of paraspinal muscle-sparing approach.

## CONCLUSIONS

The paraspinal muscle-sparing approach and the minimally invasive percutaneous approach both achieved comparable clinical outcomes to the conventional posterior midline approach in the treatment of TCBFs. All three techniques significantly improved imaging parameters, facilitated neurological recovery and demonstrated favorable safety profiles. However, compared with the posterior midline approach, the minimally invasive approaches offered notable advantages, including shorter DoO, reduced IBL and faster postoperative recovery, making them promising alternatives for clinical application.

### Authors’ Contributions:

**BT:** Designed this study and prepared this manuscript. **ZL** and **FL:** Collected, analyzed clinical data, are responsible for the accuracy and integrity of this study. **TL** and **QL:** Critical Review, Analysis. All authors have read and approved the final manuscript.
